# Wireless Technologies for Social Distancing in the Time of COVID-19: Literature Review, Open Issues, and Limitations

**DOI:** 10.3390/s22062313

**Published:** 2022-03-17

**Authors:** Sallar Salam Murad, Salman Yussof, Rozin Badeel

**Affiliations:** 1 Institute of Informatics and Computing in Energy, University Tenaga Nasional, Kajang 43000, Malaysia; salman@uniten.edu.my; 2 Department of Network, Parallel & Distributed Computing, University Putra Malaysia, Seri Kembangan 43400, Malaysia; rozinbabdal1987@gmail.com

**Keywords:** COVID-19, pandemic, social distancing, wireless technologies

## Abstract

This research aims to provide a comprehensive background on social distancing as well as effective technologies that can be used to facilitate the social distancing practice. Scenarios of enabling wireless and emerging technologies are presented, which are especially effective in monitoring and keeping distance amongst people. In addition, detailed taxonomy is proposed summarizing the essential elements such as implementation type, scenarios, and technology being used. This research reviews and analyzes existing social distancing studies that focus on employing different kinds of technologies to fight the Coronavirus disease (COVID-19) pandemic. This study main goal is to identify and discuss the issues, challenges, weaknesses and limitations found in the existing models and/or systems to provide a clear understanding of the area. Articles were systematically collected and filtered based on certain criteria and within ten years span. The findings of this study will support future researchers and developers to solve specific issues and challenges, fill research gaps, and improve social distancing systems to fight pandemics similar to COVID-19.

## 1. Introduction

Because of COVID-19, worldwide perceptions of the pandemic disease have been transformed, having clear implications for global health and the economy. Within six months of starting in Wuhan, China (from January to June 2020) [1], a total of 210 nations and worldwide have confirmed more than ten million infected people, including more than five hundred thousand deaths, according to the world health organization (WHO) [2], COVID-19 has caused significant economic losses in addition to the worldwide health catastrophe (e.g., a potential 25% rate of unemployment inside the U.S [3]. In March 2020, one million Canadians lost their jobs [4], 1.4 million jobs were lost in Australia in the year 2020 [5], in addition to an estimated global Gross domestic product GDP loss of 3% [6], an economic downturn, as many experts had expected [6,7,8]. Solutions are urgently needed to stop the spread of the disease, lowering its harmful effects and buying more time for pharmaceutical solution development in such a situation. Severe Acute Respiratory Syndrome (SARS), Influenza A virus subtype (H1N1), and COVID-19 are examples of contagious diseases where social distance can be an efficient non-pharmaceutical way to limiting the spread of disease [9,10,11].

So-called “social distance” refers to measures that work to prevent disease spread through minimizing human physical contact frequency and intensity, including the closure of public spaces (e.g., schools and offices), avoiding large crowds, and maintaining a safe distance between individuals [9,12]. Because it reduces the likelihood that an infected person would transmit the illness to a healthy individual, social distance reduces the disease’s progression and impact. During the early stages of a pandemic, social distancing techniques can play a crucial role in decreasing the infection rate and delaying the disease’s peak. Consequently, the load on healthcare systems is reduced, and death rates are reduced [9,10,11].

It was shown that the effects of social distancing techniques on the number of instances as reported, on a daily basis [13]. It can be seen that social separation can help minimize the highest number of cases [11] not overburden public healthcare resources. In addition, social isolation can help lower the number of affected people in the long run [11].

**Figure 1:**
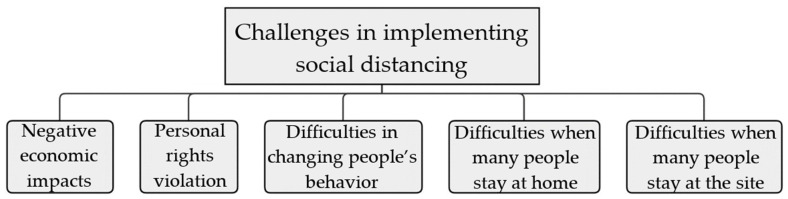
The biggest obstacles to implementing social distancing.

The earlier social separation is put into place, the more powerful the effects will be [13]. A number of countries have introduced social distancing measures, including travel restrictions during the current COVID-19 outbreak, shutting public areas and making public warnings they should retain a spacing of 1.5–2 m once they walk outside [14,15,16]. Such strong and large-scale initiatives, however, can be difficult or even impossible to undertake, e.g., individuals still need to go outside to get food, health care, or important work because not all public spaces can be blocked. In such context, technologies play a key role in helping social distancing procedures. For example, they work by sensing the distances between people and notifying them when they get too close to each other. Methods of social distancing could be divided into two categories: public and individual. For the public, this means closing or restricting entry to educational institutions and workplaces, cancelling large gatherings of people and placing travel restrictions on them as well as border control and quarantining structures. As far as personal precautions are concerned: isolation, quarantine, and encouraging people to maintain physical boundaries between them [12]. A country’s social distancing policy usually takes effect after it has been implemented (e.g., lockdown at diverse stages) for 13–23 days; as observed in the second graph, the daily number of new cases begins to decline as social distancing is enforced (i.e., flattening the curve) [17,18]. Considering the enormous possibility, social distancing is ultimately successful when it is used appropriately. However, as demonstrated in Figure 1, it is not an easy task to implement because of several challenges.

Travel restrictions, border controls, and the closing of public spaces are among the social distancing measures that negatively affect the economy. This could lead to the authorities removing restrictions too soon, e.g., Iran, South Korea, China, Germany have to re-impose limitations after lifting restrictions too early [19,20]. Restrictions like quarantines, cancelling mass meetings, and solitary may contradict cultural and moral precepts, e.g., Iran locked religious amenities throughout lockdown [21]. Aside from that, tracking people’s movements and tracing their contacts is essential, e.g., contact tracing in Singapore [22], also violate people’s privacy. As a result, people may not adhere to these rules. People find it difficult to cope with social distancing, even when they desire to. There are many challenges, such as determining and keeping a safe distance of 1.5–2 m between people all the time, health care and food must still be obtained from other sources, and working from home is not always feasible (essential workers). Many individuals will be forced to work or study remotely due to the closure of schools and companies, which will result in a massive rise in Internet usage and online service needs, e.g., Zoom users [23] and Microsoft Teams users [24] have increased up to 1270% and 775%, respectively, while there is a lockdown. Some systems may have issues when implementing social distancing strategies in specific contexts. Examples include tiny system capabilities and small space systems designs. Accordingly, many strategies and concepts have been presented in the literature deploying several types of wireless technology in various settings to combat COVID-19 dissemination through social distancing techniques.

The motivation behind this research is that different research articles reviewed certain aspects related to social distancing using wireless technology such as (i) application, architecture, and/or security of the internet of medical things, (ii) Opportunities, challenges of emerging technologies, (iii) future smart connected communities to fight COVID-19 outbreak, and (iv) contact-tracing features and architecture. In addition, few studies, such as [25,26], have presented a comprehensive survey of enabling and emerging technologies and briefly explained fundamentals and characteristics of different technologies to fight COVID-19 pandemic. However, to the best of our knowledge, no study in the three databases Institute of Electrical and Electronics Engineers (IEEE), ScienceDirect, and Web of Science (WoS)—the studies exist in databases and sources that are given in Section 2 which are explained in the exclusive and inclusive criteria—has examined and analyzed all the existing studies related to social distancing using wireless and emerging technologies to identify and investigate the issues, challenges, and limitations in the existing systems and models. Therefore, this study surveys and highlights these factors which need to be addressed by prospective researchers and developers from the literature, this research comprehensively introduces and studies these concerns.

This paper is organized in five sections as follows. Section 1 presents introduction including background on social distancing, the importance of it, and challenges implementing social distancing approaches. Section 2 contains methodology including studies selection and numbers of articles from the search results. Section 3 shows in detail Background on wireless technologies for social distancing and taxonomy of literature that shows and explains important factors such as scenarios, implementation methods, and technologies being used. Section 4 includes considerations on issues and challenges, in both, general issues in wireless technologies and in models of existing studies. Section 5 presents limitations and weaknesses of wireless technologies, and models and methods found in the literature. Finally, Section 6 covers summary of this research work.

## 2. Methodology

Many creative approaches have been launched recently as a result of the rapid growth of emerging technologies, which can generate favourable conditions for social distancing. This section explains the primary search, download, and filtering parameters for the papers included in this research. Another important aspect in this section is how studies were selected based on the eligibility criteria. Various search results are examined, as well as article types and numbers found. Then, a thorough taxonomy is offered, and finally, a discussion includes issues, challenges, weaknesses and limitations are introduced. A query search setup is made up of three libraries, namely WoS, IEEE, and ScienceDirect. All social distance research, including wireless technology, were included by these databases, which were deemed sufficient and appropriate.

### Method, Study Selection, & Search Results

Databases, keywords, and criteria are only a handful of good features and settings used for this study to collect the relevant articles. An essential part of any study is identifying research areas by using appropriate keywords. Include “social distancing” and “wireless” when searching for existing studies on social distancing involving wireless and/or emerging technology. We only looked at English-language publications. From 2011 through 2021, every paper included in this review process was published within ten years. Even though social distancing has a wide range of applications and domains, studies that focus on pandemics and/or COVID-19 are rare and focused. As a result, all research that focused on social distancing concerning the COVID-19 pandemic were included in this research.

**Figure 2:**
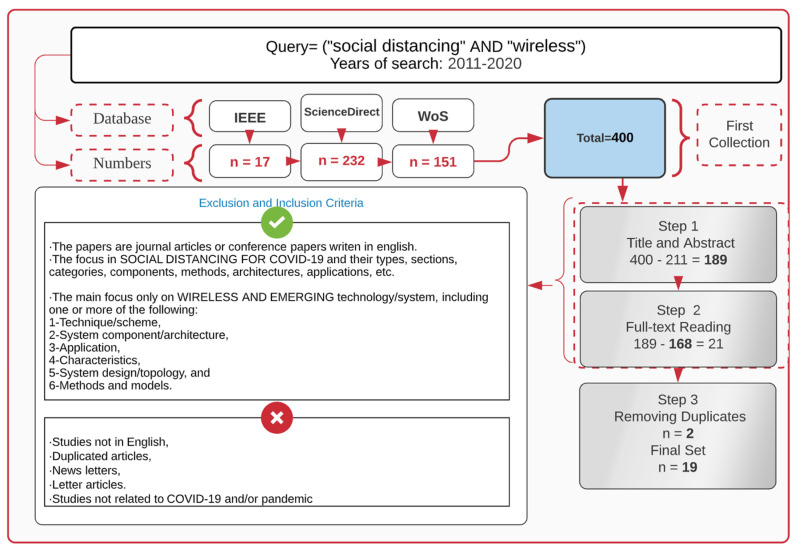
Development study selection, including search query, inclusion criteria, and exclusion criteria.

Figure 2 summarizes all of the steps, elements, inclusion and exclusion criteria, and stages included. To assess each article for various qualities, data was collected and extracted into an Excel spreadsheet, then used to list and arrange the articles into matching categories. In order to find articles, the first step was to do a keyword search on three databases. The initial query search resulted in 400 articles from all databases. A three-stage filtration process was used. There were 221 unrelated papers that were excluded in the first round. Following a full-text reading of the remaining 189 papers, 168 were removed, resulting in the publication of 21 articles.

**Figure 3:**
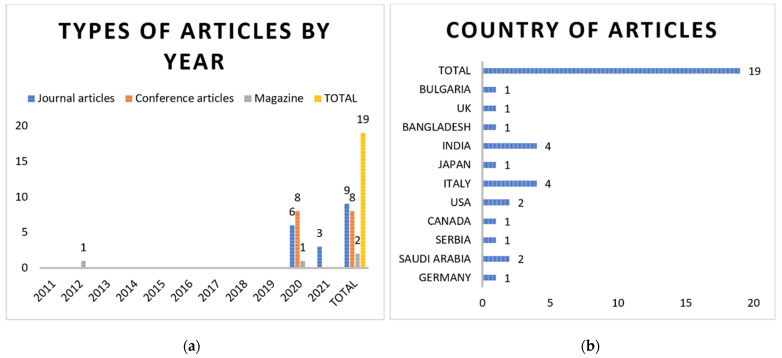
(**a**) Number of articles’ types based on their year of publication, and (**b**) Number of articles by country of origin.

Two papers were ruled out for being duplicates in the last stage of this process, leaving 19 final publications that were considered relevant to this research in it is totality. Figure 3a presents the number and types of articles by year of publication as well. In addition, the country distribution of the final set of papers is shown in Figure 3b.

## 3. Wireless Technologies for Social Distancing

### 3.1. Background

The concept of social distancing may not be as easy as physical distancing, given the rising complexity of viruses and the fast expansion of social interaction and globalization. It encompasses numerous non-pharmaceutical activities or efforts designed to reduce the spread of infectious diseases, including monitoring, detection, and alerting people. Different technologies can assist in maintaining a safe distance (e.g., 1.5 m) between persons in the adopted scenarios. There are a number of wireless and similar technologies that can be used to monitor people and public locations in real-time.

To collect valuable data, such monitoring is necessary (e.g., records of people inside buildings, contacts, symptoms, crowds, and social distancing measures violations) to facilitate social distancing. Technologies like Bluetooth, Ultra-wideband (UWB), Global Navigation Satellite Systems (GNSS), and thermal sensors allow you to track the movement of infected persons, as well as the contacts that they established. They can take precautionary measures if they’ve been exposed to infected individuals (e.g., self-isolation, limit access, and test for the disease).

**Figure 4:**
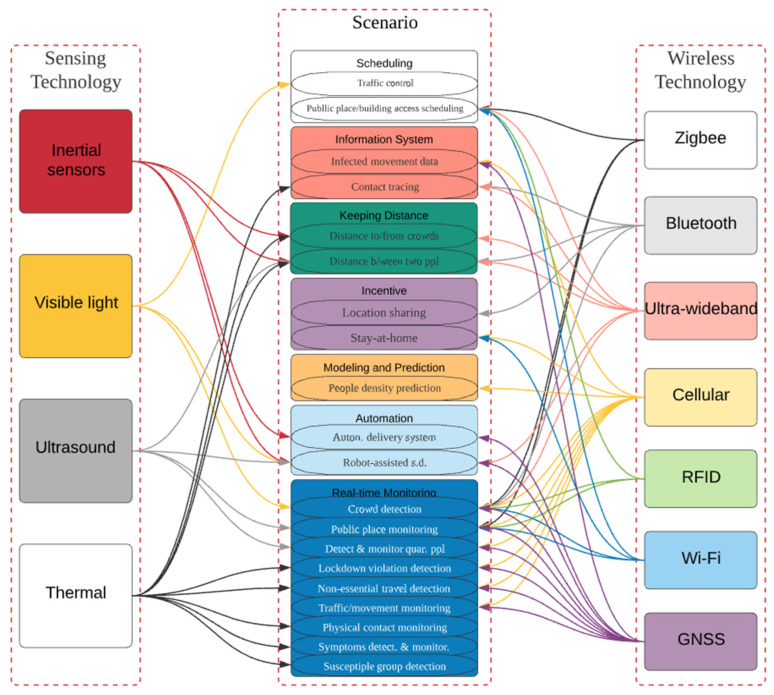
Applications of wireless technologies to different social distancing scenarios.

Furthermore, technologies such as Wireless Fidelity (WiFi), Radio frequency identification (RFID), and Zonal Intercommunication Global-standard (Zigbee) can be used to schedule access to a specific building. It’s possible to acquire pandemic data using the latest technologies. Infected and susceptible people can be predicted based on the data. Figure 4 shows the applications of technologies to specific social distancing scenarios introduced and discussed in [25,26].

As shown, some technologies, e.g., Cellular, Global navigation satellite system (GNSS), Artificial intelligence (AI) and Thermal, can be used in various scenarios, while technologies such as Zigbee, RFID, Visible Light (VL) and Ultrasound are appropriate to fewer scenarios. Scenarios that belong to the same group are all the same colour. Same-coloured arrows connect one technology to multiple scenarios. In this section, we will discuss the features and characteristics of the models and designs, which consist of deploying various wireless technologies used for making successful social distancing scenarios. The focus will be on issues and challenges in terms of system building/or system functionalities.

### 3.2. Taxonomy

A taxonomy of relevant articles from the literature is presented in this section for the first time in the social distancing research area. The taxonomy classifies all social distancing studies into many vital categories. Our taxonomy consists of vital aspects that are important to review and clarify the details that are required for creation of any sort of social distancing research using wireless technology.

Many types of social distancing scenarios were employed in various situations. Real-time monitoring and keeping distance were the most scenarios used so far, while scheduling and incentive are the least. Specifically, the research [27] temperature and saturation level monitoring using Message Queuing Telemetry Transport (MQTT) was designed. This study covers the use of MQTT to classify patients based on temperature and saturation levels measured.

The study [28] looked at how to interpret body-induced thermal signatures for physical distancing and temperature screening. A statistical model was used for the proposed framework to capture body-induced thermal signatures from noisy data, and a mobility model is used to detect multi-body activities and minimize erroneous target detection. Furthermore, body-induced impacts fade practically exponentially with the range between the Infrared sensors and the person. Whenever the user is in close proximity to the Infrared radiation (IR) sensor array, the temperature is automatically detected. The study presented a stochastic method for thermal indicators that is less susceptible to such flaws. The suggested screening approach is based on a mathematical model and Bayesian decision concept, and it allows the user to navigate during the screening procedure whereas its location is estimated (constantly) by using Bayesian framework. This approach can be used as a general framework for multi-sensor installations and big IR arrays.

An Internet of Things (IoT) solution for social distancing in smart cities relying on multi-sensor was described in [29], and the goal of this study was to offer an IoT system founded on an IoT, wireless sensor network (WSN), and algorithms (Neural Network (NN), and Shortest Path First (SPF)) that can distinguish warnings, accessible exits, gathering points, safest and shortest routes, and congestion based on real-time data collected by sensors and cameras using computer vision. The IoT system was used to tackle two distinct problems: an urgent scenario and a surveillance situation. Data, both raw and processed, is transmitted to a platform interface for further live monitoring of environmental and architectural factors.

The authors of [30] utilized AI and a mass surveillance system, as well as a Beyond fifth-generation (B5G) framework that takes advantage of the 5G network’s low-latency, high operability, to diagnose COVID-19 using chest X-ray or computerized tomography (CT) scan images, and to establish a mass surveillance system to monitor social distancing, mask wearing, and body temperature. A user layer, an edge layer, and a cloud layer form the framework. The COVID-19 detecting approach suggested here could be applied to any contagious diseases. As a result, it will aid in reducing hospital overcrowding, verifying non-COVID-19 patients, and processing critical personal data at the edge to preserve privacy.

Research [31] focused on the assessment of technology-assisted distance estimation for safe aircraft boarding. It proved the normal COVID-19 distance estimation’s vulnerability to faults, which could also lead to huge inaccuracies in distance calculation and the impracticability of typical tracking methodologies during passenger boarding/deboarding. It reviewed and assessed the limitations of received signal strength indicator (RSSI)-based distance estimate in difficult circumstances, and it proposed the use of additional signal measures and to optimize technology-assisted social distancing effectiveness. The optimized consideration of passenger groups in the context of a pandemic boarding scenario significantly contribute to a faster process (reduction of boarding time by about 60%) and a reduced transmission risk (reduced by 85%), which reaches the level of boarding times in pre-pandemic scenarios. The associated cabin and the linked position sensing paradigm will be a major enabling technology in this regard. Path-loss (PL) modelling in these circumstances is highly reliant on the propagation environment and must frequently be calculated empirically. The PL exponent is the sole tuning parameter available, and it is very volatile for diverse scenarios and cannot explain propagation processes in different contexts in a universal way.

The study [32] proposed a model for spotting violations of distance. The paper introduced an IoT edge-based solution focused on model transformation and an entire weighted graph for detecting breaches of social distance standards in indoor public settings. The system configuration allows for the capture of the status of individuals in a group based on predetermined distance measurements. The system examines the distances among group members and determines how much the social distancing metric is being violated in a given area. The proposed IoT edge-based architecture of the system is made up of numerous elements, each of which is in charge of accomplishing and administering a variety of duties. Wearable instruments and WSN infrastructure (sensor layer), data handling at edge points (middleware layer), and a cloud graph repository are among the elements (data storage layer). The suggested system includes a notification delivered to an authorized person alerting them of the status of physical distancing measures at a specified moment and recommending when immediate intervention is required. The mathematical basis of the described system is a comprehensive weighted graph model with a limited network capacity made up of groups of persons representing a real-world crowd in an indoor context. Indoor tracing, positioning, localization, and observation systems are a set of networked devices that are used to locate or track items. Individuals inside buildings, train stations, airports, basement garages, and other sites sharing characteristics of systems that identify violations of distance measures are included.

The study [33] featured surveillance and prediction for mass gatherings (MGs) throughout geographic borders. An integrated platform of this type may assist in the early detection of infectious disease risks of global value, as well as offer information into which diseases are more likely to expand into the MG, support with anticipatory monitoring at the MG allow mathematical modelling of infectious illness transmission to and from MGs, simulating the impact of community health actions directed at various local and global stages, serving as a foundation for scientific study and development in MG health, and improve interaction between the scientific sector and stakeholders on a national, international, and worldwide scale. MGs are conceptualized as global-to-local-to-global occurrences since they consist of a reasonably balanced global-to-local convergence followed by a local-to-global separation of people from all over the world.

The study [34] primarily utilized AI for prediction. It processes users’ medical data in real time to forecast COVID-19 infection by watching their symptoms, and it promptly sends an emergency alert, medical reports, and major warnings to the user, their guardian, and doctors/experts. It gathers sensitive data from hospitals/quarantine sites via patient IoT devices in order to take the required actions/decisions. Furthermore, it broadcasts a warning message to official health organizations in order to limit the spread of chronic illnesses and take rapid and timely action.

On the other hand, the studies that considered keeping distance have similar factors but different scenarios. Specifically, the study [35] introduced an intelligent wearable device, Suraksha, that may be worn when walking outdoor and will aid sustain social distance. Some exist devices cannot capture motion in all directions; however, the suggested gadget can recognize motion in all directions in 360 o up to 1.5 m. As a result, the user does not have to be concerned about their surroundings at all times. It is a small gadget that is uncomplicated to operate and is constructed using basic electronic parts. The device can also connect to health applications through Bluetooth and allow contact tracing. The effort is motivated by the necessity to develop a gadget that allows people to keep a safe distance from others during the transmission of contagious diseases. The design is tailored towards every individual. The described system is a low-cost, effective, and lightweight means of assisting individuals in maintaining social distance by sensing close movement between individuals and activating an alarm in case of tight proximity.

The research [36], created a system for measuring social distancing in university campuses leveraging microcomputer modules, with mobile hubs provided to students as entry permits. The data gathered by mobile nodes is forwarded to a monitoring center. The node can be worn around the neck with the help of a neck strap. Distances among students are calculated by transmitting and receiving Bluetooth low-energy (BLE) advertising packets between nodes on a regular basis. The findings revealed that using average or median RSSI data, it is conceivable to approximate distances generally, and RSSI varies based on the direction of the person who wears the device, and also the sender-node battery power has no effect on RSSI.

A protocol for physical distancing on another wearable device proposed in [37] named “6Fit-A-Part”. The study focused at on the creation of a wearable gadget that generates an alarm if another sensor of a similar type is identified within a certain distance. It makes use of off-the-shelf UWB wireless technology to estimate distance with others in the area in real time. They created a one-to-all ranging mechanism that can reliably estimate distance to surrounding devices and alert the user if the distance drops below a predefined threshold in a minimal period of time. Whenever physical boundaries occur between devices, the equipment may adapt for human occlusions and eliminate unwanted cautions.

The research [31] conducted an assessment of technology-supported distance gauging approaches in an aircraft cabin setting by means of a radio propagation simulation constructed on a three-dimensional aircraft model. It has revealed the conventional COVID-19 distance estimation’s sensitivity to mistakes, which can lead to massive mistakes in distance calculation and the difficulty of typical tracing methodologies during passenger boarding/deboarding.

The study [38] investigated the feasibility of using indoor localization technology to determine the distance between users in indoor areas. The research also investigated how information about people’s connections obtained can be leveraged at three stages: beforehand, during, and post individuals access a service, as well as several concerns that concretely deal with the real-world deployment of an Indoor Localization System (ILS). A standard design for an ILS with three illustrative use-cases was also proposed. The authors offered a framework for evaluating the functionality of the proposed architecture.

Various kinds of implementation were used in the literature using different tools and settings. On one hand some studies have made their implementation using real experiments such as [27,30,35,36,39,40,41], while some studies have conducted a simulation-based implementations, such as [29,31,32,33,34,42,43,44,45]. On the other hand, few studies made both implementation, simulation based and real experiments, such as [28,37], while the study [38] did not present implementation method.

Some of the systems and techniques in the literature as shown in the taxonomy, require internet access to be fully functional; for example, internet connection is needed when using a web-based indoor navigation system in [41], while using a low-cost portable device to maintain distance as in [35] or using video surveillance for real-time distance estimation in [40] do not need internet access to store or retrieve information for further processing; however, we found the almost equivalent number of studies used both approaches.

Another factor taken into account by our taxonomy is the usage of systems and techniques found in the literature, specifically, some approaches are compulsory to follow at the location and others are not, for instance, when utilizing certain scenarios such as contact tracing in [43], individuals must follow the procedures imposed by the system at the site and take action by interacting with some components included in the system such as smartphone, while in [45] using machine learning for COVID-19 cases prediction, it is not compulsory for people to take action or to be a functional part of the system components.

Location of the existing systems is another important factor, whereas some studies considered the indoor area while others focused on outdoor. Utilizing social distancing systems inside buildings and in close areas found to be easier and more controllable, and more important than outdoor social distancing approaches because it is harder to minimize physical contact and monitor in confined areas and small size rooms such as aircraft cabins. The study [35] proposed solution makes use of passive infrared sensor (PIR sensor) sensors which detects humans and animal bodies and not arbitrary objects, the proposed wearable device is more suitable for outdoor area, and the study [41] presented an efficient and cost-effective indoor navigation system for driving people inside large smart buildings to preserve distance among people, whereas the study [40] presented a simple real-time method for distance estimation with any kind of camera between people applicable either in close quarters or open spaces. On the other hand, it is hard to determine the suitability of location for some studies.

**Figure 5:**
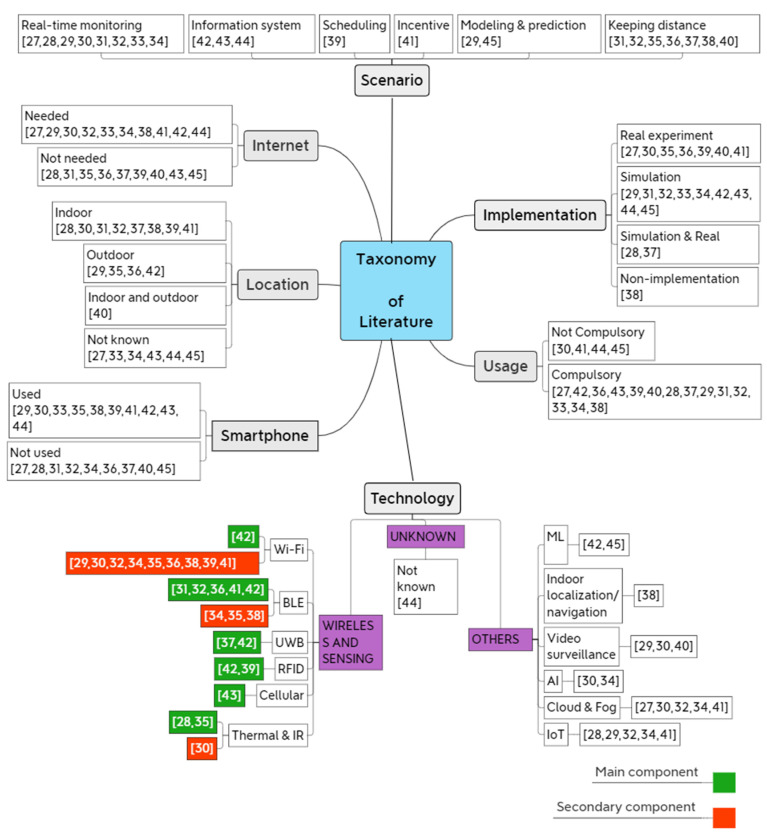
Taxonomy of literature.

It is worth notice that smartphones are essential for some approaches such as contact tracing, Bluetooth-based and WiFi-based systems, while others do not require users to have a smartphone such as temperature screening or distance monitoring using a thermal camera. The most important factor in our taxonomy is the type technology used by previous studies, including sensing components, wireless components, and other components. Various wireless and other technologies have been used in the previous social distancing systems and scenarios; however, some systems consist of using specific technology as the main component, for example, using IR to detect the distance between two people, while others use additional wireless technology as a secondary component as supplementary for other functions included in the system such as for uploading data for backup. For wireless and sensing technologies, the most technologies were used in the literature are WiFi and Bluetooth, authors [42] used WiFi as main component in their study, whereas other studies have used the WiFi technology as secondary component for their proposed system, such as [35,36]. The Bluetooth technology has taken a significant role in many studies as main component such as the indoor navigation system in [41]. Moreover, only few studies have considered the use of other technologies as main component in their systems, such as UWB, RFID, Cellular, thermal and IR. In addition, for other types of technologies such as Cloud, IoT and video surveillance, were the most used in the literature, followed by Machine learning and Artificial intelligence, and finally indoor navigation was considered by one study. All the abovementioned factors and studies are summarized in the taxonomy as shown in Figure 5.

## 4. Issues and Challenges

There were a few concerns, challenges, and limits in the suggested systems in most social distance studies that used various types of wireless and upcoming technologies with the considered method, environment, and/or specification. The technology attributes being employed, the planned system, model and/or design, and the surroundings are a few of these considerations, and other factors are taken into account as shown in Table 1.

Accuracy and privacy are the two key considerations when it comes to social distancing apps and scenarios. Most research has focused on accuracy as the most critical aspect.

Another component of accuracy is the rate of lost data, or the strength and condition of the connection, which a variety of variables can cause. The system would be more reliable to use in real-world applications with increased accuracy.

In addition, there are two types of privacy taken into account for the social distancing systems: (i) Privacy of data, and (ii) privacy of people’s information. In particular, the level of privacy (if existed) in some systems could be estimated as low and high as stated in Table 1. When it comes to privacy, using any system that requires sensitive information makes people uncomfortable, especially when the system asks for sensitive information, such as:

- **Location:** Users’ location is required by some systems and applications, such as wireless services and Global Positioning System (GPS) systems.
- **Symptoms:** Many suggested research would involve taking vital signs of persons and/or patients without their knowledge or agreement to gather data.
- **PRV:** Some systems may require people to respond to specific discoveries and/or decisions made by the systems they are using, such as denial of access, blocking, and the like.

**Figure 6:**
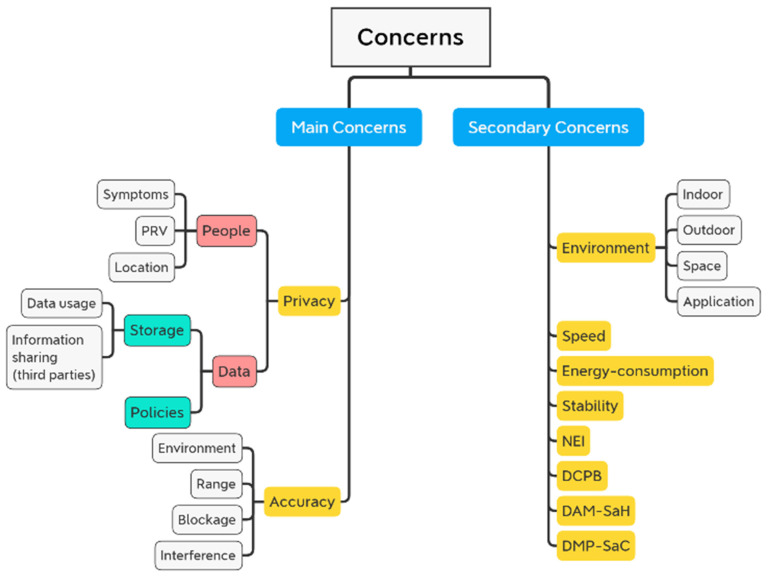
Main and secondary concerns in social distancing scenarios while using wireless and emerging technologies.

Concerns about privacy revolve around where the acquired data will be stored and who will have access to it, and systems and mobile applications’ policies, which users must agree to in order to use them. The main and secondary factors, including accuracy and privacy, are summarized in Figure 6.

### 4.1. General Issues & Challenges

**Figure 7:**
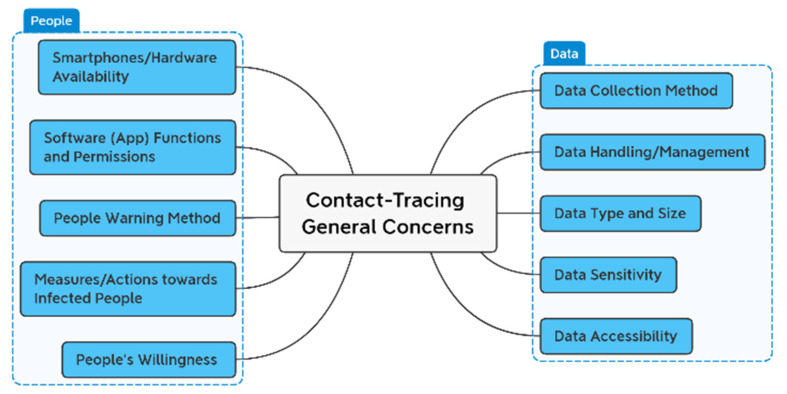
General concerns in app-based contact-tracing scenario.

Globally, there are more than three billion smartphone users [46]. In five years, smartphone users are predicted to climb by one billion [47]. It is, therefore, possible to track down individuals using mobile phone apps, but the difficulty is how to safely handle the gathered data and what information should be collected [48]. Additionally, people’s willingness to install apps and the availability of cellphones in all locations within a community are also significant obstacles to be overcome. Once the data has been collected, the next logical question is how to properly alert individuals of the risks. Figure 7 shows the concerns in app-based contact tracing.

As a result of the factors shown in Figure 7, it is still challenging for the contact-tracing apps to overcome the concerns in addition to the client density (increased numbers of users) and distance among them. Erroneous positive/negative warnings of such apps, together with the privacy considerations previously discussed, discourage their everyday use. So, the technology used by the apps determines their success on a large scale. Applications that rely on WiFi/Bluetooth signal strength, for example, often fail in crowded situations or surroundings with obstructions and impediments.

To notify people of the likelihood of infection or to track the likely infected person’s contact trajectory, various technologies are presently being considered, including contact tracing, narrowcasting, broadcasting, and so on [49]. Ensuring timely exposure warnings relies on accurate contact tracing data. It is necessary to share personal information about the users, such as their location, who they contacted, etc. The app can only deliver accurate data if the information it has collected is sufficient. The downside is that more information leads to more significant privacy violations, a big problem in today’s society. It’s essential to understand how smartphone applications work to recognize the privacy problems associated with them entirely.

The mechanism applied in the Bluetooth-based contact tracing app has been questioned by the authors in [50], such as, whether or not a user’s privacy can be protected from the app provider, prohibit snoopers from accessing personal info with the app, the authority’s response if they are approached after knowing their information, and so on. Because of this, in a pandemic crisis, discovering the infected or possibly infected person is not enough; also vital is how to educate them or what measures should be done to restrict them. As a result of varied societal standards, measures that look effective for one country may not be effective. The Cable News Network (CNN) reports that was considered the Health Code app [51], which is utilized in many places of China, works as follows: users of the app are asked about their symptom and travel history, the possibility of exposure to COVID-19-positive individuals, their workplaces, housing addresses, cell phone numbers, passport information, national identity number, etc. will be checked. A Quick Response Code (QR Code) will be provided to the mobile phone after authentication. It can be either red, green or amber in color. Fourteen days will be required for users with a red code to be quarantined by the government or self-quarantine, amber code users will be quarantined for 7 days, whereas green code users are deemed risk-free. Someone purposefully providing false information about their trip plans, symptoms, or being in close touch with a COVID-19 positive patient will receive a green code and will likely infect many individuals before they are caught, which is the fundamental flaw of this application.

In addition to China, South Korea was one of the first countries to be infected by the new coronavirus. As a precautionary measure, the South Korean government placed all foreign passengers in self-quarantine. They were forced to use a self-diagnosis app and update their health condition on a regular basis during the quarantine period so that the government could track down any potential symptoms [52]. All individuals who had direct touch with the travellers were also put to self-quarantine and were subjected to the same surveillance procedure. In this case, however, the main issue arises when the acquired data is shared with multiple entities, such as the police department and health insurance companies, central government agencies, health care professionals, health care associations, and others. As a result, it violates data privacy regulations. Because of this, governments around the world have established measures such as shelter in place or stay at home [44]. However, universal isolation policies damage the fabric of society by eliminating social interactions, limiting care for people with disabilities, and increasing mental health issues. On the economic side, such policies reduce productivity, interrupt logistics providers, and cause financial markets to tremble.

For MGs, disease surveillance can be conducted both locally and worldwide. Still, there is a lack of integration with the two levels of epidemiological data that can be obtained from such sources. There are many challenges associated with integrating real-time intelligence from global surveillance systems with information regarding international movement between reported disease outbreaks and MG sites when it comes to disease surveillance during MGs [53,54]. Obstacles in reporting systems and multilateral coordination remain despite implementing WHO’s updated 2005 International Health Regulations [55]. For example, countries that perceive economic or other concerns may be unwilling to rapidly and thoroughly release information regarding the hazards of contagious diseases that are of concern to international populations. Despite the fact that internet-based informal data sources are becoming increasingly crucial for worldwide surveillance of infectious illnesses, there are still significant obstacles to overcome [56,57]. These methods have a number of inherent limitations, including the inability to separate background internet noise from significant public health indicators, false alarms and the need to validate signs of public health relevance (accuracy). This disadvantage may be mitigated by aggregating many data sources so that assessments are not reliant on a single source of information. It is also necessary to address the digital gap in future systems development in order to provide a consistent and comprehensive global coverage of infectious illnesses. It’s possible for planners to get information on participants’ geographic origins because some MG participants must register or obtain a visa in order to attend (e.g., city-level vs. national level). Other data sources (ticket sales for sporting events) could provide further information regarding the local and global environments from which spectators are coming. However, estimating how residents would interact with other MG participants can be challenging because some may be drawn to MG-related events while others may be inclined to avoid them.

It’s not known how many people are infected with a very mild sickness or even undetectable infection and how contagious they are. For every recorded case, assumptions range from 5 to 50 people without symptoms. Current estimations of the COVID-19 outbreak are not accurate, and it will take months or possibly years to collect all the necessary data. To determine how many people are genuinely affected, every country shall do major testing on the population. This information, however, is neither available now nor was it available at the time of the epidemic. Obtaining useful insight into the likely spread of the virus and the mortality toll depends on the accuracy of the outbreak prediction model. Also, the unique coronavirus outbreak was markedly different from prior outbreaks in a number of ways, which raised questions about whether or not the existing models could give appropriate predictions and results in the real world. When it comes to the COVID-19 viral outbreak, many unknown elements still impact its spreading. These include population behaviours that are complicated and vary from country to country, as well as government responses to the virus outbreak, such as declaring a state of emergency. The performance of current models had been seriously hindered by these unknown variables [58]. The influence of social distancing, quarantine, and curfew has been incorporated into some of the more modern models for predicting outbreaks, i.e., [59,60].

For data protection, the access control (AC) system used in [38], only authorized users can view protected material, and they have access to the authorization levels necessary to complete their activities. Despite the importance of AC systems, their integration into the localization system architecture remains a difficulty, especially in light of the General Data Protection Regulation (GDPR). We agree with this idea and believe that a lot of effort must be put into integrating diverse technologies that enable the identification of persons both indoors and outdoors. When it comes to pandemic preparedness, such efforts will determine the consequence. Individuals can more easily determine if a situation is unsafe or not in open places, such as a supermarket. On the other hand, individuals need assistance with automatic tools when working in indoor and confined areas. Social distancing systems must migrate from manual to automation systems, and this requires technology that can lower deployment costs. As an example, let’s take a look at those systems that count the number of persons in each room. To limit access to such sites, the system informs arriving visitors after a particular density has been reached. Anyhow, even if no precise localization system is utilized, common interfaces must be researched in order to connect with all end-users. Another factor to consider is that the number of persons who make reservations to visit a specific setting can be calculated. Obviously, however, it really is an approximation that is susceptible to a variety of unexpected circumstances. To verify the crowding status and perhaps receive notifications, a user interface is required. When it comes to the IoT system for social distancing and emergency management in smart cities using multi-sensor data [29], people’s behaviour is difficult to predict since they may try to avoid the sensors. The distance in such a setting could also affect the accuracy.

There are two major drawbacks to the mass surveillance system-based healthcare framework [30], which employs multiple deep learning (DL) algorithms. First, local authorities will have issues because of the need for a vast COVID-19 dataset that includes numerous variables. Second, the health care sector and other stakeholders must embrace and clarify the conclusions of deep learning before they can be acknowledged, as well as other contributors. The real-time epidemic monitoring and control system faces a number of challenges, including the need for vast amounts of pathological, radiographic, genetic, and other types of epidemic-related information to be absorbed, stored, processed, and classified based on large amounts of COVID-19-related data. There is a need for real-time support for human physicians at the COVID-19 treatment facilities on the front lines and faster diagnosis, which means processing time is shorter and less time-consuming. The accuracy and energy consumption of wearable IoT devices, as well as the technology used to communicate with them, are also essential aspects to consider.

For proximity tracing, most current techniques rely on the Google Apple Exposure Notification (GAEN) framework, which uses BLE-based distance calculations [61]. A BLE measurement’s precision, on the other hand, raises serious questions [62]. Due to Non-line-of-sight (NLOS) and multipath signal reception issues, wireless ranging suffers from variations in signal parameter measurements, which can lead to distance errors.

The study [35] has developed a low-cost, easy-to-use wearable device that uses Bluetooth signals to measure distance among people. It was anticipated that the wearable device would work, but the study did not take into account whether people would be willing to use it. Aside from that, the study do not take into account storing the obtained data for backup or analysis. Both centralized and decentralized BLE-based tracing methods have serious flaws. First, it is not possible to track someone if they do not have a smartphone with Bluetooth connectivity. The majority of people in a first-world country like Singapore or Germany use cellphones, yet not everyone has Bluetooth enabled on their device at all times. Second, despite the fact that Bluetooth technology is regarded as a low-cost, reliable, and energy-efficient alternative, any unauthorized user can access the information contained in mobile devices over the Bluetooth link [63,64,65]. According to the study [64], employing mobile Bluetooth technology for android smartphones poses security risks. The authors of the study [66] examined the iOS platform’s security risks. Data privacy is, therefore a key problem when it comes to Bluetooth-based apps.

In railway systems, people tracking relies on travellers-tracing technologies such as WiFi, RFID, Bluetooth, and UWB, when wireless cellular network signalling and GPS are either unavailable or limited [42]. Managing pedestrian flows within stations, mainly underground or at airports where ventilation is critical, is difficult in the real-time estimation of the distance between people and/or objects utilizing video surveillance [40].

Massive and unobtrusive screening of individuals in public places is a vital duty for the temperature screening system employing thermal vision in [28] to ensure safety in congested shared spaces and help early non-invasive diagnosis and reaction to disease epidemics. Typical thermal vision issues include the transient disappearance of subjects [67] due to noisy readings and external heat sources that could be misinterpreted as false targets. Temperature measuring by contactless devices [68,69] is gaining popularity since it allows for automatic screening of large groups of people [70,71,72]. To provide continuous real-time monitoring, existing automated systems require users to be less mobile. As a result of the lack of a consistent processing interface and tools for thermal camera scanners, contactless instruments like these have some limitations in terms of temperature precision. Temperature measurement with thermal cameras has been investigated in a number of research [73,74,75]. However, these devices’ mass adoption is hindered by their autonomy, size, and expense.

Numerous IoT indoor localization solutions also use RFID-based wearable devices [76,77,78,79]. Wearable device Identities and data gathered by the linked sensor can be transmitted to an RFID reader across a distance of 100–200 m via radio frequency signals. For example, in [78], researchers proposed an active RFID-based indoor locating system that uses a frequency-hopping mechanism to decrease the acquired RSSI fingerprinting data distortion. Two types of low-cost tags are used with a tag–tag communication protocol: virtual reference tags and object tags. Reference tags are spread throughout the covered area to improve accuracy, while object tags deliver RFID positions to RFID readers. However, two issues can arise when adopting RFID-based systems: the signals can be intercepted by unauthorized individuals, and RFID tags can be readily cloned.

In the study [36], when adopting an ID card tapping against the RFID reader method, students touch their ID card and create physical contact with the equipment, which implies it may induce infection transmission even if individuals do not have any evident symptoms of COVID-19 illness.

The technology that serves as the foundation for position identification is a critical issue for indoor navigation. The RSSI is transformed into a distance value in [34] to locate an overlapping spot by creating two or more circles or other ellipsoids. Fingerprinting [80] is another traditional technique for indoor localization that was used with Bluetooth beacons. They deployed 19 beacons distributed across 600 m^2^ that could achieve localization accuracy of less than 2.6 m in 95% of the cases.

**Figure 8:**
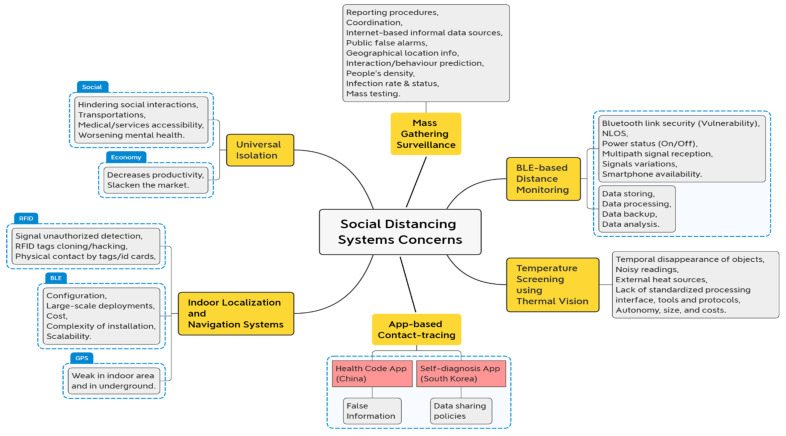
Main General issues and challenges of social distancing systems.

Despite the impressive results, fingerprinting is dependent on empirical mapping of radio signals and therefore requires it for each floor plan configuration, rendering it unsuitable for large-scale implementation. While numerous advancements and novel methods have been attempted to enhance indoor navigation using technology, there are still many hurdles ahead for adoption, such as those stated in the introduction (cost, complexity of installation, scalability). Aside from proximity-based positioning, there are two more techniques: symbolic and absolute positioning. Absolute positioning, such as GPS, determines the precise location. It can be determined using the triangulation method, and the results are coordinates. The accuracy of GPS depends on the approach; in open areas, GPS can provide 1m of accuracy for citizens and roughly 2 cm for military use. In terms of accuracy, symbolic positioning falls somewhere between absolute and proximity-based positioning. Unfortunately, GPS signals are poor in enclosed spaces and underground. Figure 8 summarizes the above issues and challenges of social distancing systems.

### 4.2. Issues and Challenges in Method, Model, and Design

Mobile users equipped with smartphones can get directions to their location using an effective and cost-effective web-based indoor navigation system [41]. Consider, for example, a patient in the hospital who has to arrive to a given ward on a certain date. To get directions to the destination, he/she can utilize a web app on his/her smartphone. An ideal web app for this study would consist of the following features:

(1) In order to do this, it should be able to detect and monitor the user’s location within the building,
(2) It should also be aware of the surrounding environment,
(3) The system should be able to determine the optimum route between the user and destination, taking into account the measure of interest (e.g., the shortest path, the most extensive way and/or passage, the lowest people density level, and so on), and
(4) While viewing a map and offering spoken or iconographic instructions, it should guide the user to their desired destination.

Despite its limitations, the Proximity Estimator component tells you if a searched device is within range of another device. When it comes to installing beacons inside a building to aid indoor navigation, there is no universally acknowledged approach. As a result of the fact that all BLE Beacons interfere with one another for the duration of the navigation, using additional Beacons may not always be financially feasible and does not always lead to better positioning accuracy. On top of all that, even when using optimal Metal Oxide Semiconductor (MOS) (to analyze user experience), we can still witness a tiny improvement in coverage because there are areas where not all BLE Beacons conflict with each other. Because the user must keep their phone on all the time, they must also utilize the app constantly.

It can be concluded from the study [42] that the more the volume and movement of user equipment (UEs) captured by travellers-tracing enablers, the greater the number and mobility of individuals in various age groups can be provided and recommended accordingly. There are minimal difficulties for offered recommendations to age-group travellers using secure mobility sequencing against COVID-19. Time to guarantee the time-specific mobility for age-specific travellers would be allocated for safeguarding vulnerable age-group from others. Off-peak day travel time slots would be 10 a.m. to 12 noon and 2 p.m. to 4 p.m., with instructions posted at stations. This is a great way to save money and time. Staff can be asked to help in this regard, or travellers-tracking capabilities on smartphones can be used to send alerts.

According to [43], two scenarios were taken into consideration when gathering location data utilizing the automated contact tracking approach using geolocation data from mobile-cellular networks. Firstly, it is necessary to collect data only from a single Mobile Switching Centre (MSC) owner if an infected individual travels inside the coverage area of more than one Base Station Controller (BSC). Secondly, if they move between many Mobile Switching Center (MSCs) owned by different service providers, the authorities will need to gather all travel data. There are various security risks associated with using centralized databases, including a man in the middle (MITM), Structured Query Language (SQL) injection, distributed denial of service (DDoS), etc. But these dangers are common to many centralized systems, so it’s not surprising. While there may be privacy issues for non-users when users and non-users are linked via social relationships, this is true with other contact tracing techniques as well. Unintentional effects can also occur when an individual is confirmed with COVID-19 positivity, as can their family and friends. Issues of permission are addressed from the perspective of the carriers, enterprises, and end-users of the system under consideration.

Tracing people using traditional methods like phone interviews and record-keeping has limitations in terms of scalability. The number of people who adopt (i.e., download) the app and the percentage of people who self-isolate according to an exposure alarm make it problematic in the Advanced Automated Contact Tracing (AACT) presented in [44]. Digital contact tracing cannot be successful without both of these factors. It would be adequate to have reduced adoption rates if all users self-isolate in response to an exposure notice; however, a lower reaction rate to alerts calls for a higher general population adoption rate. To be successful, AACT relies on users’ willingness to follow recommendations as well as user adoption. AACT would have a limited effect on disease spread if people did not self-isolate in response to notifications. Also, exposures cannot be tracked at reduced adoption rates, which reduces the results.

AACT has the most chance of success if it is universally adopted and universally responded to by everyone. There was evidence in the study that even with low adoption and response rates, it is possible to dramatically reduce disease spread while also minimizing the number of people who are separated. SARS-CoV-2 transmission from exposed persons to other susceptible people was not considered in the model because of the small number of people who paid attention to the warnings. Both AACT and shelter in place [41] produce excellent outcomes in terms of lowering infection cases.

However, such a policy would confine more than 71 million people at its peak compared to isolating around 12 million individuals in AACT to accomplish a comparable decrease. Furthermore, we dispute the study’s offered scenarios by asking the following questions: (i) How can we ensure that everyone has access to the internet? (ii) If they do and have installed the app—which is a complicated process for the entire population—how can we ensure that everyone is constantly checking their phones to respond to warnings, specifically when an alarm is triggered? Furthermore, as previously said, contact tracing apps demand persons’ location, which abuses their privacy.

To ensure safe aircraft boarding during the COVID-19 Pandemic, distance measuring research was conducted in [31] that focused on the environmental and equipment side. They found that environmental circumstances have a greater impact when there is a lack of available space, as well as concave and metallic surroundings, such as in airline cabins. Natural reflections, dispersion, and attenuation make these surroundings difficult for wireless signals to operate in. By displaying a ray-tracing simulation of likely signal routes leading to poor reception in an Airbus A321 passenger cabin in their research. Additional attenuation occurs as a result of passengers’ communication behaviour, which influences radio behaviour and reduces the correlation between measured RSSI and estimated distances. Only by fine-tuning a so-called PL exponent can RSSI-based distance take environmental factors into account. When it comes to social distancing applications, even small changes in parameters might have huge effects. Consensual parameter selection results in shorter measured distances, which accelerates the probability of false-positive tracing. Exponents that are too small for the PL component can result in positive ranging biases and thus increase the rate of false-negative occurrences, resulting in breaches of undetected distance. A key challenge is to develop a universal parameterization for a time-invariant and dynamic environment. When using the RSSI-based distance estimate scheme, there may be a decorrelation of RSSI with distance because environmental implied errors can be changeable [81]. This instability can lead to undetected infection chains or higher false-negative encounter rates, diminishing overall effectiveness and user satisfaction in social distancing and contact tracking scenarios.

**Figure 9:**
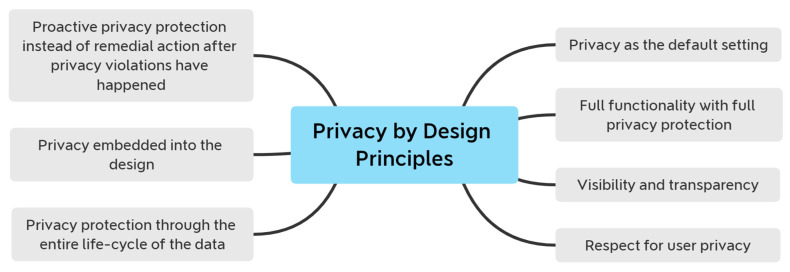
Privacy by design principles for contact-tracing apps.

The potential loss of control over personal data obtained by location-based services is a severe issue. We can use the contact tracing apps developed to combat the COVID-19 epidemic as an example. Users are concerned when this happens since they did not anticipate the data they provided to be used for commercial purposes. The issue of ensuring the privacy of data gathered by an ILS was discussed in depth in [38]. Figure 9 illustrates the seven privacy-by-design principles that should be respected.

**Figure 10:**
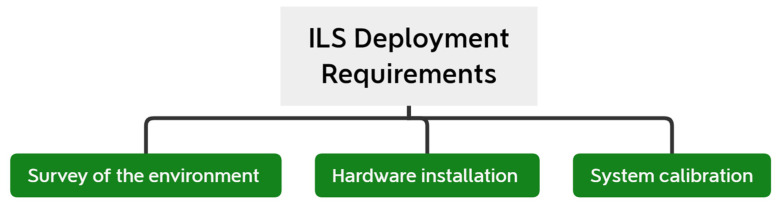
Important required steps for ILS Deployment.

Strategies for applying these ideas into the creation of social restriction modelling systems. Parallel to this, privacy-by-design measures such as data reduction should be taken into consideration. In addition, we believe that information exchange, active defense, and automation approaches should be well integrated. According to the study’s findings [38], an orthogonal component of privacy was examined, namely the ILS’s reputation for trustworthiness. It requires implementing various protection procedures and the assurance that no personal information is leaked at any step. Managing the system’s trust also involves ensuring the confidence of third-party elements that an ILS can incorporate. As illustrated in Figure 10, to deploy an ILS, you must complete the three aspects listed.

Furthermore, the environment’s shape affects signal propagation. Because there are fewer constraints in open areas, wireless signals are likely to reach further. Finally, the presence of outside spaces that need to be covered has an impact on overall performance. The previous stage leads to the installation of the ILS’s necessary hardware. Before deploying anchor nodes that allow users to be located, such as WiFi access points, Bluetooth tags, or UWB boards, it is needed to find locations where they may be deployed. When deploying hardware, it is generally necessary to have a nearby power source, no adjacent barriers, and a comfortable distance from end-users. The combination of these conditions makes deployment difficult in locations that are not intended for it.

Thermal signatures created by the body are used for physical separation and temperature screening in [28]. The detector array receives raw thermal IR pictures structured as 2D frames of 8 × 8 pixels (M = 64). Each IR array sensor’s Field of View (FOV) is structured into a grid consisting of multiple physical Regions of Interest (ROI). As the distance between ROIs grows, more than just the body is measured; everything else inside that ROI, including the background, is often measured. Furthermore, the real-time prediction of the distance between persons and/or objects via video surveillance in [40] shows that the estimated accuracy is within a statistical error of 2 to 3 cm.

According to the developers of the temperature and saturation level monitoring system utilizing MQTT in [27], MQTT requires the presence of connectivity between a client and a broker rather than among clients. The CPU use is determined by the data transport rate. It is also influenced by the type of data sent and the other operations that are running concurrently. The latency encountered diminishes as the number of publishers increases, resulting in energy and bandwidth conservation.

The proposed system for monitoring social distance utilizing microcomputer modules and BLE at university campus setting in [36] uses signals from the university WiFi network to estimate the location of nodes on campus in an approximate manner, which could hinder the accuracy. The method utilized to alert people is heavily depending on the RSSI signal received, and this signal can be influenced by the movement, resulting in decreased accuracy, even between the identical orientations, the RSSI varied. The average and median RSSI, on the other hand, dropped with a change in direction.

The fascinating wearable low-cost IR-based device termed “Suraksha” in [35] can be worn and/or removed at the user’s discretion at any time, based on our analysis of its setup and functioning principle. Since its light emitting diode (LED) light only blinks when other persons are spotted, and there is no indication that the gadget is on, users can disable it while it is still being worn. As a result, not everyone will follow instructions exactly. Whereas the chances of infected persons tricking the system into spreading the illness are high, there’s a lot of potential for inaccuracy. Additionally, according to the research, “An alarm is also transmitted to their mobile phone application via Bluetooth,” indicating that the phone and Bluetooth in it must be on at all times, adding to the whole system’s complexity and power consumption. Another problem with their model is that the authors failed to consider the possibility that the sensors on the gadget could measure the wearer’s body temperature. For example, if they raise their hands to pick up items from higher shelves or lower their heads to tighten their shoes, this could lead to erroneous results.

In [39], a distributed Framework employing RFID is proposed for the smart cart, with a restricted waiting time. A person’s waiting time in a distributed system is essentially nonexistent. Waiting times grow tremendously under the current system. Due to the growth in the number of shopping cards, this technique will require an increased number of devices, resulting in an increase in cost.

The use of solar cells to recharge the batteries in the sensing node box was presented in [29] as an interesting IoT system for social distancing and emergency management in smart cities that uses multi-sensor data. However, because the sensing node is powered by solar cells, it is dependent on clear skies and may encounter problems if it rains or clouds. As a result of the system’s large number of nodes and thus high-power consumption, using a power supply source of this type poses a number of issues in terms of keeping the system working smoothly. Because of the reliance on solar-cell recharging, the battery may run out of charge, resulting in an unsustainable electrical supply for some or all nodes. As a result, there’s a chance it’ll deliver inaccurate information or operate too slowly. Another issue with this method is that all users must keep their smartphones on because the WiFi camera is designed to provide notifications to users’ devices. Furthermore, implementing this system across a greater region necessitates the use of more sensors, increasing the overall cost while maintaining accuracy.

Doctors have utilized CT scans to evaluate patients’ lung health because COVID-19 harms respiratory epithelial cells. CT scans and X-ray imaging equipment are practically standards in all hospitals. Therefore, they might be utilized for COVID-19 screening. CT scans and X-ray image processing utilized in [30] have the drawback of requiring radiology expertise and spending more time, which is problematic for patients who need a COVID- 19 diagnosis right away. As a result, an automated evaluation model must be designed to save healthcare experts’ valuable time while also serving more patients [82]. The study [30] highlighted the use of mobile phone apps to collect and upload important indicators, including temperature, heart rate, blood pressure and cough quality. Still, they did not demonstrate how cellphones are capable of such precise measures. In addition, the cloud used throughout the study has servers with significant compute power made up of a bunch of Graphics Processing Units (GPU).

The strategy presented in [34] for predicting and preventing COVID-19, which uses cloud/fog computing linked with AI, can assist in controlling this virus outbreak at a low cost. The potential for patients to synchronize their information to the healthcare repository placed in a fog unit, which can contain an entire copy of the data from the remote server, is the essential key element of cloud storage. Furthermore, to the best of our knowledge, the COVID-19 side effect data collection is not fully available and/or acknowledged in any official repository such as UCI, CDC, NHS, and so on that may be used immediately for analysis. Despite the fact that no data set could be found on the internet, all probable cases of COVID-19 infection were identified, and a new set of data was prepared.

An NLoS and NLoS with a reflection for radio propagation were discussed in the research [31]. As a result, two primary concerns for technology-aided social distancing in demanding environments like the aircraft cabin or public transportation were identified: (a) dominant path prediction and (b) time resolution. However, under settings with a low signal-to-noise ratio, the direct path may not even be recognizable from the present noise, thus leading to incorrect distance estimates. The Channel Impulse Response (CIR) characteristics disclose a superposition of distinct signal routes within just a few nanoseconds, which is notably difficult for narrowband technology because time resolution is dependent on existing bitrates. The 2.4 GHz band has a usable bandwidth of 83.5 MHz for use with BLE. The sample rate is equal to the bandwidth inversed, or 12 ns. Seven rays reach the receiver in 2 ns in the case shown. The conical shape and metallic shell of aircraft cabins are similar to those of buses and metro trains in terms of signal propagation environmental factors. Therefore, similar effects should be predicted. Differences in the built-in antenna properties across various manufacturers and devices make it difficult to estimate distance using RSSI values. Typical WiFi and Bluetooth antennas on smartphones have a great degree of directionality, which results in different antenna gains depending on the phone’s orientation. As a result, the distance measurement may be incorrect by some proportion depending on the relevant orientation of the device. External impacts on signal transmission must also be taken into account. The use of the received signal strength (RSS)-based range and the resulting low accuracy resulted in a number of difficulties. For the most part, there is no way to tell how RSS factors affect user devices because they are so varied between manufacturers, generations, and hardware configurations (especially antennas). Because the embedded antennas do not broadcast in a consistent pattern in all directions, the device’s orientation does have a significant impact on RSS. This has the potential to lead to substantial differences in distance estimates. The PL resulting from environmental dependencies is a major factor for RSS-based distance measuring in addition to device-related issues. PL modelling’s usefulness depends on the absence of obstructions or other interruptions in the first Fresnel zone, which is a rotating ellipsoidal region between the transmitter and receiver where most of the signal energy is conveyed.

Nevertheless, multipath consequences damage this assumption in real-world applications, particularly in indoor areas. Reflections and scattering contribute to signal energy dissipation, phase and time shifts of the signal as it travels to the receiver, giving the signal several pathways to the receiver. After considering all possible propagation pathways, the RSS can be estimated as a combination of time- and phase-shifted signals as well as signals with varying levels of amplitude and attenuation. When calculating the PL in these circumstances, careful consideration must be given to the propagation environment, and the results are frequently based on actual measurements. However, PL increases logarithmically with distance in all derived models, though. Multipath reception has also been linked to RSS fluctuations due to shadowing, according to previous research [31]. Multipath effects can cause RSS changes of up to 5 dB even in static conditions [81]. As a result, it’s been demonstrated that PL follows a log-normal random distribution (normal distribution when measured in dB) assuming a fixed distance, which therefore impacts the RSS at the receiver [83]. A heterogeneous propagation behaviour will result from additional attenuation produced by objects and structures; in other words, increasing density reduces accuracy. PL fluctuations can be seen as a result of both constructive and destructive interference. If you look at the center, there are fewer obstructions, therefore the signal power drops less quickly than at the margins. The implications of insufficient environmental modelling errors are significant for the presented application domain of RSSI-based contract tracers, since they lead to unreliable tracing outcomes. While the visible RSSI may be steady, the resulting distance faults strongly correlate with the actual distance between transmitter and receiver. As a result, it is impossible to extract a universal statement on RSSI assessment failure for distinct configurations resulting in a specific distance estimation error. Additionally, the PL exponent also has an impact on the estimation error. However, typical mobile devices (like smartphones) do not deliver extra information to determine propagation variations. A more detailed investigation of signal propagation routes is required to improve range accuracy even further, as RSSI-based distance estimations have the central issue of being unable to identify or cope better with multipath effects.

There was a model transformation strategy for determining distancing breaches in weighted networks proposed in [32], Neo4j nodes indicate persons (individuals) in a crowd, while undirected and weighted edges reflect the exact distances between individuals at the corresponding time point. As a result, the graph shown at a particular point in time depicts how the social distancing rule is being applied. In the actual world, the person node contains characteristics that show the position of a human who is wearing a sensor or is connected to a communication equipment. People’s actions in the actual world can be unpredictable at times, which can influence calculations and thus affect accuracy.

Prediction of worldwide movements of bystanders to MGs presented in [33] is essential to public health since this crowd is often quite large and regionally diversified, and hence potentially be an excellent source of infectious disease introduction to the MG. Although modelling traveller movements from their global origins to MGs is difficult, worldwide passenger-level airline statistics have been utilized to estimate the quantity and international origins of individuals travelling to Vancouver, BC, Canada, before the start of the 2010 winter Olympic Games [54]. Since each population’s geographic origins, modes of transportation, and infectious disease burden may vary substantially, it is crucial to study their travel movements independently to estimate public health concerns. There are many domains where vital data is kept. Still, it can be tough to link them all together to create the synthesis you need (e.g., host governments, international organizing committees, and transportation industries).

However, even though infectious disease surveillance and modelling are aimed at different local and global levels, it is often not well integrated into a single framework for vulnerability monitoring and evaluation, which limits its ability to produce valuable public health intelligence in near real time. People’s movements at MGs appear to be impacted by the common exogenous variables, hence clustering of individuals in space and time is anticipated in crowd behaviour models, might be affected by social interactions and herding effects. They are significant factors of clustering in location and time; and usually lead to congregation and hence crowding towards a few focal spots.

**Figure 11:**
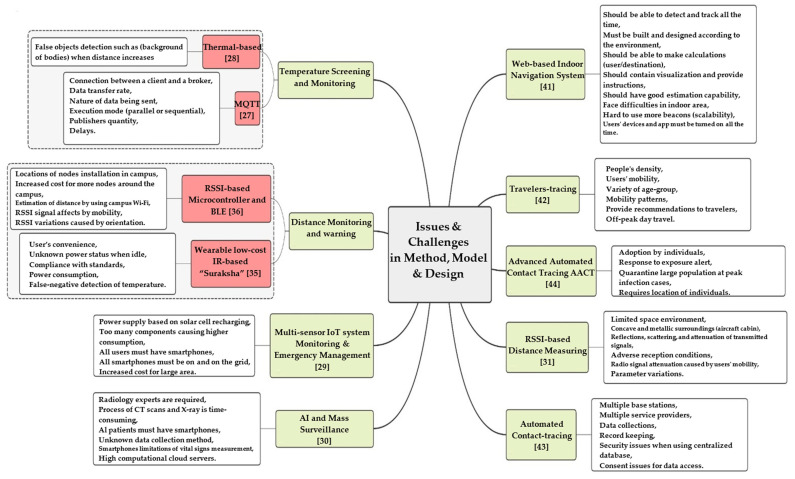
Issues and challenges in method, model and design.

It’s worth noting that the suggested algorithm CESBAS in [45] failed to account for the significant increase in new cases for the date 17 February 2020 which was considered in their study for the COVID-19 cases in China from 21 January 2020 till 18 February 2020, retrieved and merged from WHO reports, resulting in somewhat lower metrics. This is expected behaviour; in any machine learning system for time series prediction, there will be an inaccuracy owing to unpredictable external factors: (i) Reducible, and (ii) Irreducible error influence prediction accuracy. Compute time is also included in comparative analysis. However, these metrics cannot be fairly compared since the research claimed that the very same dataset was acquired and simulated under equivalent experimental settings of algorithms given in [84]. Yet, the approaches have been evaluated on various computing platforms. Nevertheless, Our World in Data shows that between 16 February and 17 February 2020, there was an even greater surge in new instances (from 51 k to 70 k). Due to this, the authors intended to examine CESBAS-ANFIS and BAS-ANFIS with this dataset as well, in case it affected prediction accuracy; this process was used in [45] to assess the resilience of the CESBAS-ANFIS and BAS-ANFIS frameworks. There are numerous concerns and hurdles to overcome while doing social distancing research using wireless and developing technologies, as highlighted in Figure 11.

## 5. Limitations

### 5.1. General Limitations of Wireless Technologies for Social Distancing

In this section, the limitations of wireless and emerging technologies are summarized. In order to enable, encourage, and compel people to exercise social distancing, WiFi technology is a popular approach. As WiFi technology evolves, interference may compromise the accuracy of using WiFi. Furthermore, in other places like hospitals, utilizing WiFi might be forbidden in specific areas. Furthermore, WiFi-based technology is primarily employed in indoor areas because it necessitates the usage of many access points for localization, which is not always possible in outdoor settings.

One of the most promising technologies to helping with social distancing is cellular technology. It helps keep track of quarantined or infected people. However, the usage of user location data for social distancing practices raises serious privacy issues among citizens.

Bluetooth technology has the potential to enable social distancing. However, users’ privacy must be considered because the services demand the sharing of information with authorities and third parties. This might be a significant research topic to help protect privacy while also encouraging people to share their medical information to help stop the transmission of infections. The accuracy of localization techniques when users’ equipment is kept inside pockets or bags and the necessity for users’ devices to always be in Bluetooth mode are also disadvantages of Bluetooth technology in the social distance that must be taken into account. Additionally, merging Bluetooth with other technologies to enhance the accuracy of localization is an open research area.

The IR-UWB systems can be considered a fantastic solution for social distancing for indoor and outdoor scenarios with the aforementioned potential uses. Other RF technologies like WiFi or cellular do not demand the installation of additional hardware for tracking reasons and can be used to monitor the location of self-isolated people to see if they are violating quarantine regulations. Phones like the iPhone 11 series that support UWB can let users practice social distancing without location and navigation features. This approach, however, only works with a newer iPhone that has a UWB chip.

While the GNSS-based service has several benefits in the practice of social distancing, e.g., tracing users, preserving distance and group surveillance. However, it has several drawbacks that limit the range of situations in which it can be used. This service necessitates tracking users’ locations in real-time via GPS, increasing deployment costs and creating privacy concerns for users. Furthermore, the accuracy of GNSS services is generally low when it comes to measuring the distance between two individuals, especially for distances under two meters. As a result, newer advanced GNSS technologies can be employed to boost GPS accuracy, such as the global navigation satellite system GLONASS augmentation system based on pseudolite [85], the differential GPS positioning methods: differential position, differential pseudo range and differential carrier phase [86], the enhanced calibration method of GLONASS inter-channel bias for GNSS real-time kinematic (RTK) [87], and the enhanced RTK positioning algorithm [88].

Despite this, such technologies are still extremely costly and have yet to be broadly used in public services, demanding even more research in this area.

When a pandemic breakout occurs, Zigbee technology can help enable social isolation by isolating people from each other. On the other hand, Zigbee is a new technology that has not yet found widespread use in our daily lives, limiting the range of possible applications. Nevertheless, the number of Zigbee-enabled devices is likely to overgrow soon, thanks to backing from major firms like Amazon, Google, Apple, and Texas Instruments [89].

In order to permit social separation, RFID technology may be the answer. On the other hand, RFID has not been extensively accepted since it’s so tough to place into effect. Individuals must wear RFID tags to use RFID technology to track their movements. On the other hand, RFID tags are not as widely available as WiFi access points or Bluetooth devices. As a result, there are just a few practical applications for RFID technology in social distancing. Moreover, there are many types of RFID tags, some of them can be easily duplicated. Therefore, the real entities in the system are vulnerable to being fraudulent.

Ultrasound can be used in a variety of social distancing settings. Ultrasonic positioning system (UPS) systems like AB and CK can be used to locate and alert people to keep their distance in keeping distance scenarios. Furthermore, because of its restriction characteristic, ultrasound is one of the most efficient technologies with binary positioning, which is very important for observing and estimating the number of individuals in the same room. Ultrasound may help navigation for UAVs and healthcare robots in automation scenarios, primarily in the indoor area.

Smartphones with inertial sensors have made it possible to construct positioning systems based on INS. INS positioning devices, especially for walkers, can play a significant role in distance keeping scenarios since they are widely available. INS-based approaches can help improve the effectiveness (more accurate path and lower travelling time) of existing navigation systems in different settings, such as medical robot navigation and UAV delivery. However, utilizing inertial sensors outdoors is preferable since it provides better accuracy, whereas indoors, it must be paired with other technology to lower the error rate.

We can use industrial ways to establish a crowd surveillance system on a broad scale in shopping malls or superstores and other public locations, such as airports, railway stations, and hospitals, using the existing illumination infrastructures. Facility administrators can promptly alert or notify users in a crowd (e.g., varying the lights’ colour temperature in the high-density zones). On the other end, assistance systems serve to minimize the number of employees/volunteers, nurses within public facilities or to eliminate the close contact between consumers and patients. When combined with other radio frequency technologies like Bluetooth and Infrared, the location-based applications are not disrupted even when the user is not constantly using the smartphone (e.g., the phone is in the pocket).

The Visible light communication (VLC) technology may provide connectivity between the intelligent traffic control system and automobiles operating in the outdoors. However, its principal drawback is that interference from surrounding, and solar light substantially affects the VLC channels [90,91]. As a consequence, RSS-based positioning techniques and outdoor communications weaken. Moreover, smart retail systems prove that VLC technology outperforms other RF technologies regarding indoor localization and navigation quality [92].

**Figure 12:**
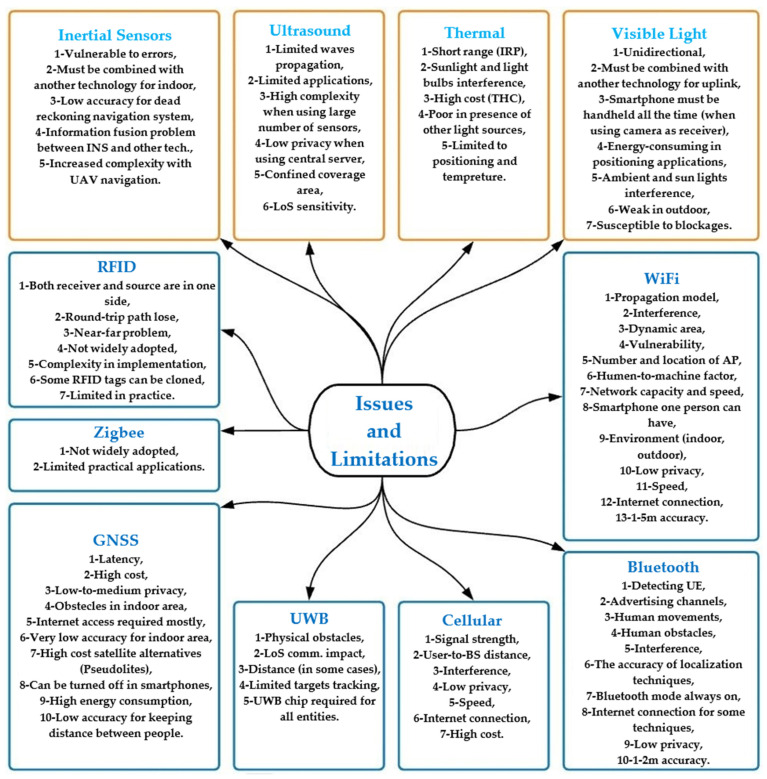
Limitations of wireless and sensing technologies for social distancing.

Finally, thermal-based solutions are effective in social distance scenarios, especially when there is limited light. The IRP can be employed for location and tracing in short-range communications. Due to their high range, some light-weight thermal imaging camera (THC) devices can be used for real-time tracking across vast distances. However, when adopting THCs in practice, the high expense of THC should be considered. The difficulties and limits of the technologies discussed in this section are summarized in Figure 12.

### 5.2. Limitations of the Method, Model and Design

The models and designs in the literature that consisted of focusing on various social distancing scenarios have several limitations and/or missing factors. In this section, we analyze the existing systems and models and identify their limitations and weaknesses.

By warning the user when someone is identified within close range, the low-cost gadget presented in [35] can successfully be used to maintain the minimum physical distance required by social distancing guidelines. However, the PIR motion sensor used, on the other hand, may struggle in a dynamic setting and hence cannot ensure accuracy. Moreover, the functionality of the proposed device might be bound by the height of the device and the height of the intended objects to be detected. For instance, when a tall person wears the device in the head, it may not detect short people around. The effective management of power distribution across the nodes within the system has not been assessed yet. However, they have not proved the approach or mechanism for delivering and receiving this data. The study claimed that the information obtained can be provided to a smartphone without the need for registration or the use of an app. It’s also risky to send and receive data without registering because it opens the door to unreliable sources providing incorrect information. Finally, there was no mention of the system’s capacity or the environment in the research.

With temperature and saturation information from the study [27], the researchers deployed MQTT to evaluate patients, but they did not specify how the temperatures and saturation levels were measured or what sensors were employed.

The main objective of the study [42] was to identify high-risk age-group travellers utilizing passengers-tracing enablers, allowing them to be prioritized for future monitoring and risk evaluation. The study introduced a new strategy to monitoring daily railway passengers aged 16–59 and above 60 (vulnerable age group) by recommending travel in particular train compartments, stops and platforms. However, people under the age of 16 were not taken into account.

The research [36] presented a technique for assessing social distance on university campuses. However, the investigation’s findings into the distances at which nodes may obtain BLE advertising packets in the suggested model showed that transmitter nodes could provide BLE advertising packets to reception nodes from as far away as 20 m. Consequently, it is impossible to tell regardless of whether people are in close proximity, even if they receive a BLE advertising packet. Additionally, the results were collected under restricted conditions, and further assessments such as node combinations, person variations, postures of people wearing trackers, and the environments in which measurements might be made were not taken into consideration. Because only fairly simple conditions were taken into account in the study, future studies will need to undergo evaluations in far bigger and more diverse university campus settings. Mechanisms for lowering node power usage must also be taken into account.

The study’s objective [43] was to address the stated issues in COVID-19 contact tracing utilizing smartphone apps. This study recommended employing mobile phone users’ geolocation data instead of BLE-based smartphone apps for contact tracing. However, this approach has drawbacks in that the suggested contact tracing only uses cellular networks and is therefore dependent on the availability of received back-and-forth signals. In other words, the quality of the cellular signal may be poor in certain places, such as when there is considerable interference with RF signals, and the signals may even be blocked in other areas, such as tunnels and the underground. The researchers also lacked official access to mobile user data during their investigation.

The recommended solution in the study [39] saves time by allowing individuals to bypass the crowds near the checkout counters or point of sale. However, the study stated that encryption had been used to encrypt the transmitted data, but they did not specify what type of encryption or technique was employed. Furthermore, the study does not consider problems or failures of specific components, which may increase client waiting time at the counter. When employing such systems, it is vital to consider alternative solutions because any problem or crash in the major components could result in the opposite of the intended result.

The aim of the study [40] was to alert people quickly and discretely if the proper space between them was not preserved. However, the strategy failed to account for the density and its impact on the system’s performance. In addition, the study only considered a small area.

To facilitate people’s movement in a smart city and, particularly in smart buildings, the study [41] presented a proximity-based indoor navigation system that gives access to public and private services and amenities while preventing crowding, especially in small spaces. A major flaw in this research was that it failed to take into account evaluable indicators and optimization procedures that can vary depending on building distribution, building geometry, and distancing policy.

The Processing of body-induced thermal signatures for physical distancing and temperature screening in [28] has two limitations. Body speed and movement orientations were not considered in the study, which may clearly be determined by evaluating the expected motion behaviour through time.

The interesting custom wearable device named “6Fit-a-Part” was proposed in the study [37]. Physical distancing requires additional effort to turn 6Fit-a-Part into a product, even though the study resolves the core difficulties of multi-user ranging and occlusion-aware detection. Moreover, the 6Fit-a-Part examination focused solely on a singular collision domain, i.e., all nodes may hear each other. This means Since there are overlapping collision zones, the measurement rate for such overlapping regions decreases. Since there are overlapping collision zones, the measurement rate for such overlapping regions decreases. 6Fit-a-Part is built, developed, and tested in a tiny testbed that simulates the real world, therefore, may contain some defects in accuracy.

The model has significant limitations throughout the universal shelter-in-place vs. advanced automated contact tracing research in [44]. First, they established the model with preset parameters, in real, the parameters were dynamic and varied as the epidemic developed. However, the goal of this work is to evaluate options for reducing disease propagation depending on a mutual illness concept. It is reasonable to anticipate that the comparable achievements of AACT and universal stay-at-home could be similar despite of how they were established. Second, the effectiveness of AACT could be reliant on the technology employed. Global positioning systems, for example, have less position accuracy over Bluetooth or WiFi. Whenever technology has higher location accuracy, approaches that anticipate exposure depending upon proximity between an infected individual and an application user will become more accurate (and hence affect fewer individuals). Researchers also anticipated that AACT adoption is evenly spread across the population.

Usage that is evenly distributed across a community is likely to contribute more than usage in tight regions. Furthermore, their modelling does not take into consideration transmission from infected persons to other vulnerable people (e.g., household members) between the time of infection and the time of self-quarantine. The model did not account for these kinds of third-order interactions, which skewed the results in favor of AACT. Such deviations can be minimized with widespread use, near-real-time data, and the application of self-quarantine guidelines to home exposures. In the actual world, numerous countries have begun to implement AACT to aid in the reopening of societies and the prevention of disease spreading. According to findings from Singapore, digital contact tracing shows higher sensitivity and specificity for detecting contacts than traditional techniques [93]. The information on the effectiveness of these measures, on the other hand, is insufficient and demands comprehensive investigation prior to model conclusions can be drawn. The necessity for a genuine setting is also vital considering that a variety of elements, such as technology literacy, infrastructure, regulatory laws, user adoption based on culture, and factors such as regional population migration, can all impact effectiveness. For instance, relying on the population, the probability of wide user acceptance and adherence would be reduced in the absence of government assistance. Additionally, communities with a high rate of trade with neighbouring nations, cities, or regions where AACT is not being used could overcome any AACT value. Furthermore, the usability and accuracy of AACT would be reduced in the absence of proper infrastructure (wireless data transmission systems, consolidated databases that can combine data, and so on).

In [33] infectious disease monitoring and modelling were carried across geographic boundaries, and scientific disciplines developed an integrated framework for MGs’ improved worldwide risk monitoring and risk assessment system. One drawback of this model is that estimating global travel behaviour to MGs that switch location regularly (e.g., the Olympic Games and the Fédération Internationale de Football Association World Championship) can still be challenging since previous events of travel to MGs in various locations and seasons may not be easily transferable to future MGs. The density statistic for London do not include the potential that bystanders or local citizens will change their travel arrangements to or surrounding London due to the Olympic Games. The season and timeframe of when MGs are arranged to initiate are essential in evaluating public health risks since these are typically associated with the counts and global distribution of travellers to and from the region where the MG is held and may impact the infectious interaction of viruses and bacteria with solid seasonal patterns due to the implications of weather, social, or other factors. Whereas typical and internet-based methodologies of worldwide infectious disease surveillance produce news of dozens of epidemics globally on a daily basis, in certain parts of the globe, this surveillance may lack sensitivity. However, in some others, the volume of data may burden public health end-users, who then struggle to recognize the relevance of every outbreak to an MG.

Due to the unpredictable nature of crises, an integrated fog and Artificial Intelligence healthcare framework that was introduced in [34] to predict and prohibit COVID-19 has some limitations. The proposed framework is faced with the troubles of operating in situations that bring up challenges for the use of devices customized for the regulated environment of a medical scenario. In a significant casualty incident, where physicians must deal with several injuries right away, they would be unable to react to alerts until all patients had been assessed. According to doctors, the surveillance equipment is presumed to be most successful for mapped patients waiting for ambulances. Patients that need an ambulance can be prioritized using the proposed approach. Generative Adversarial Networks (GANs) are a type of adversarial network in which GANs are generative models that use two main functions, a data generating function and an adversarial function termed the producer and the discriminator, to instantly evaluate data distributions. GAN is used in different unsupervised development models since it does not require the Markov chain to sample the input dataset frequently. In comparison to previous uncontrolled network models, the model is straightforward. Long training demands and model independence, on the other hand, have remained unchanged.

COVID-19 cases prediction by using hybrid machine learning and beetle antennae search approach in [45] has been used to carry out the optimization, but only the antecedent and conclusion variables have been considered, and a generalized bell membership function has been used. As a result, the nature of the membership function was just not subjected to improvement. According to the comparative analysis, beetle antennae search (BAS) could not even reach the right portion of the search area in specific runs due to a shortage of investigation, resulting in reduced mean values. It should be recognized that a greater dataset could have been used. Nevertheless, in the proposed scenario, comparing the effectiveness of the proposed method with other algorithms would be impossible given the fact that only a few methods, the results of which have been published in state-of-the-art journals, were executed and examined for COVID-19 case prediction. An additional limitation of the research is that everything was performed locally, offline, in this algorithm edition.

The measurement of distance among individuals in indoor spaces utilizing an indoor localization technique was used in [38] to first frame a reference architecture that could be used in various scenarios using cloud technology [94]. Unfortunately, for simplification, they did not investigate the possibilities of other Cloud-based solutions. Local maps, for instance, could be obtained from any Cloud server (e.g., Google Maps), as could a route to a destination determined by a cloud-based navigation service.

An IoT system for social distancing and emergency management in smart cities employing multi-sensor data in [29] has a few drawbacks, including the fact that the system is only appropriate for outdoor areas due to using a solar cell to charge the battery. Since the system lacks night vision for recognizing and monitoring humans, it may be challenging to sustain accuracy at night.

In [30], the interpreted AI and mass surveillance system-based healthcare framework established a mass surveillance approach to track social distancing, mask-wearing, and body temperature. However, the privacy of the patients’ important data was not taken into account in this work. The crucial data could be changed or may be incorrect. There is just a tiny amount of data about COVID-19 available in public repositories, which might be used in the anticipated AI-based technique. As a result, the system’s accuracy may be susceptible to inaccuracy.

The majority of commercially available user devices are not capable of directly measuring RSS. In terms of the environmental model, the Airbus A321 was selected in [31] because of its widespread accessibility, including its dimensions and electromagnetic (EM) model availability. Nevertheless, the researchers emphasize that the used model, both in terms of model type and degree of complexity, has a significant influence on simulation accuracy. As a result, direct proportions may be inapplicable to different aircraft cabins or application industries, such as public transportation. However, the human factor, related fundamental attenuation, or movement-induced fading were not taken into account in their simulation. While considering the complicated and tight aircraft cabin, such restrictions could result in reduced distance estimation accuracy.

The review performed for the research [32] excluded technologies and techniques associated with outdoor monitoring and tracking methods. Such approaches were deemed more sophisticated than intended for the study’s goal and typically comprise more than a single communication interface coupled to the IoT devices to contain both short and long-range communication. Delivering a detection system of distance measures with extensive coverage capabilities for heavily mobile objects was not included in their primary objective at this stage of development; the research considered depicting relationships among individuals in the crowd at not over a 4m distance.

**Figure 13:**
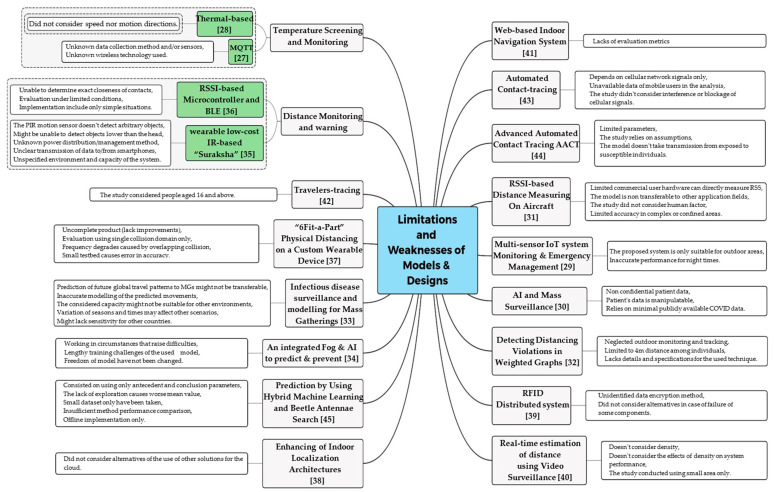
Limitations and weaknesses of method, model and design.

It should be highlighted that this research did not involve full specifications for the algorithms and approaches utilized to detect distances between persons. All the limitations and weaknesses of models and designs in the literature are summarized in Figure 13.

## 6. Conclusions

Adopting different social distancing scenarios using wireless and upcoming technologies to combat infectious diseases, particularly the ongoing pandemic COVID-19, is critical for preserving lives and providing additional time for pharmaceutical remedy development. Social distance can reduce the likelihood of disease transmission from an infected person to a healthy one by minimizing the frequency and proximity of human physical encounters, hence greatly reducing the disease’s progress and severity. In this research, we have presented a detailed survey of the social distancing, discussed its role in the current COVID-19 pandemic, and introduced various practical social distancing scenarios where the technologies can be leveraged. We then reviewed different wireless technologies to encourage and facilitate social distancing measures and presented these technologies’ issues, challenges, and limitations. A review of previous studies was introduced with a detailed taxonomy of the literature. Finally, the focus was to comprehensively examine and discuss the issues, challenges, and limitations of existing systems and models of previous studies. Such an inclusive study allows us to understand better, build, propose, and design social distancing systems using wireless and emerging technologies by prospective researchers and developers to fight COVID-19-like pandemics in the future.

## Figures and Tables

**Table 1:** Summary of issues and challenges in the literature.

Ref.			[35]	[27]	[42]	[36]	[43]	[39]	[40]	[41]	[28]	[37]	[29]	[30]	[31]	[32]	[44]	[33]	[34]	[45]	[38]
**Internal Factors**	**General**	**Approach**	MWP	VSM	TT	MWP	CT	TC	MWP	INS, ISh	PhD, VSM	PhD	MWP, CD	VSM	VSM	NK	SiP	MG	SmH	PD	IL
		**Environment**	OT	BCE	RS	UniC	G	SM	G	LSM	P	IPP	SC	SHC	AC	IPP	GB	GB, LCL	HC	NK	IPP
	**Specific**	**Accuracy**	L-M	M-H	M-H	L-M	M-H	H	M	L-M	M	L-M	M-H	L-M	L-M	M	M	M	M-H	L-M	M
		**Speed**	M	M-H	M	M-H	M	H	M	M	M	M-H	M-H	L-M	M	L-M	M	M	M-H	L	M-H
		**Privacy**	Data: H; People: H	Data: L; People: L	Data: L; People: L	Data: L; People: L	Data: M; People: L	Data: M; People: NA	Data: L; People: L	Data: M; People: M	Data: H; People: H	Data: H; People: H	Data: L; People: L	Data: M; People: L	Data: M; People: L	Data: H; People: L	Data: L; People: L	Data: L; People: L	Data: M-H; People: L	NK	Data: H; People: L
		**Energy-** **consumption**	M-H	L-M	L-M	H	H	L	L-M	M-H	L-M	M-H	L-M	H	L	NK	L	L-M	M-H	NK	L-H
		**Disconn.** **possibility**	L	L	L	L-M	L-M	L	L	L-M	L	L	M	L	M-H	M-H	L	L-M	L	NK	L-M
		**Computation**	L	M	M	L-M	L	L	M	L-M	M-H	M	L-M	H	L	M-H	L	L-M	M	H	L-M
**External Factors**	**Implementation Challenges**	**NEI**	NO	NO	YES	NO	NO	NO	NO	NO	NO	NO	L	L	H	M	YES	YES	NO	NO	YES
		**PRV**	NO	YES	YES	NO	NO	NO	YES	NO	NO	NO	M	L	M-H	M	YES	YES	NO	NO	YES
		**DCPB**	NO	NO	NO	YES	NO	NO	NO	YES	NO	NO	YES	YES	YES	NO	YES	YES	NO	NO	YES
		**DMP-SaH**	NO	NO	NO	NO	NO	NO	NO	NO	NO	NO	NO	NO	NO	NO	NO	NO	NO	NK	NO
		**DMP-SaC**	YES	NO	YES	YES	NO	NO	YES	YES	YES	YES	YES	NO	YES	YES	NO	YES	YES	NK	YES
**Terms and definitions**		**Approach**	MWP: monitor and warn people, VSM: vital sign monitoring, TT: travellers tracing, CT: contact tracing, TC: traffic control, INS: Indoor navigation system, ISh: Information sharing, PhD: physical distancing, CD: crowd density, SiP: shelter in place, MG: mass gathering, SmH: smart health, PD: people density, IL: indoor localization;																		
		**Environment**	OT: outside travelling, BCE: bandwidth-constrained environment, RS: railway system, UniC: university campus, G: general, LSM: inside large smart buildings, P: public places, IPP: indoor public areas, SM: shopping markets, SC: smart cities, SHC: smart healthcare, AC: aircraft, GB: global, LCL: local, HC: healthcare;																		
		**Specific**	L: low, M: Medium, H: high, N: needed, Nt: not needed;																		
		**External factors**	NEI: negative economic impacts, PRV: personal rights violation, DCPB: difficulties changing people’s behaviour, DMP-SaH: difficulties when many people at home, DMP-SaC: difficulties when many people at the site;																		

## Data Availability

Not applicable.
